# Open-porous magnesium-based scaffolds withstand *in vitro* corrosion under cyclic loading: A mechanistic study

**DOI:** 10.1016/j.bioactmat.2022.04.012

**Published:** 2022-04-29

**Authors:** Roxane Bonithon, Colin Lupton, Marta Roldo, Joseph Nicholas Dunlop, Gordon William Blunn, Frank Witte, Gianluca Tozzi

**Affiliations:** a Zeiss Global Centre, School of Mechanical and Design Engineering, University of Portsmouth, Anglesea Road, PO1 3DJ, Portsmouth, United Kingdom; b School of Pharmacy and Biomedical Sciences, University of Portsmouth, White Swan Road, PO1 2DT, Portsmouth, United Kingdom; c School of the Environment, Geography & Geosciences, University of Portsmouth, Burnaby Road, PO1 3QL, Portsmouth, United Kingdom; d Department of Prosthodontics, Geriatric Dentistry and Craniomandibular Disorders, Charité - Universitätsmedizin Berlin, Aßmannshauser Straße 4-6, 14197, Berlin, Germany; e Biotrics Bioimplants AG, Ullsteinstr. 108, 12109, Berlin, Germany

**Keywords:** Magnesium alloys, Bone regeneration, *In vitro* corrosion, X-ray computed tomography (XCT), Digital volume correlation (DVC)

## Abstract

The successful application of magnesium (Mg) alloys as biodegradable bone substitutes for critical-sized defects may be comprised by their high degradation rate resulting in a loss of mechanical integrity. This study investigates the degradation pattern of an open-porous fluoride-coated Mg-based scaffold immersed in circulating Hanks' Balanced Salt Solution (HBSS) with and without *in situ* cyclic compression (30 N/1 Hz). The changes in morphological and mechanical properties have been studied by combining *in situ* high-resolution X-ray computed tomography mechanics and digital volume correlation. Although *in situ* cyclic compression induced acceleration of the corrosion rate, probably due to local disruption of the coating layer where fatigue microcracks were formed, no critical failures in the overall scaffold were observed, indicating that the mechanical integrity of the Mg scaffolds was preserved. Structural changes, due to the accumulation of corrosion debris between the scaffold fibres, resulted in a significant increase (p < 0.05) in the material volume fraction from 0.52 ± 0.07 to 0.47 ± 0.03 after 14 days of corrosion. However, despite an increase in fibre material loss, the accumulated corrosion products appear to have led to an increase in Young's modulus after 14 days as well as lower third principal strain (εp3) accumulation (−91000 ± 6361 με and −60093 ± 2414 με after 2 and 14 days, respectively). Therefore, this innovative Mg scaffold design and composition provide a bone replacement, capable of sustaining mechanical loads *in situ* during the postoperative phase allowing new bone formation to be initially supported as the scaffold resorbs.

## Highlights

• First report on *in vitro* cyclic loading of MgF_2_ coated open-porous Mg scaffolds in HBSS simulating 2–3 months in humans.
• Fluoride-coating slows down corrosion under cyclic loading *in vitro.*
• Entangled scaffold structure accumulates local corrosion debris which keeps the mechanical integrity over 14 days *in vitro.*

## Graphical abstract

**Figure:**
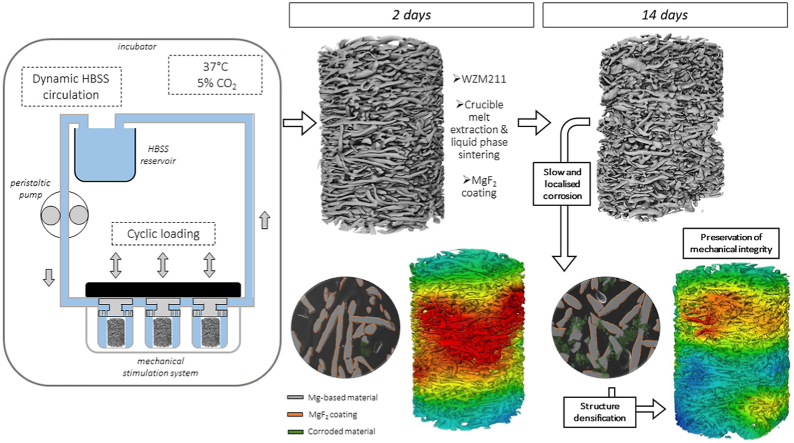

## 1 Introduction

Bone is a unique organ with the ability to self-regenerate through physiological remodelling or in response to moderate injury, without the occurrence of scar tissue [1]. However, high-energy traumatic injuries, diseases, tumour resection or osteomyelitis are extreme clinical conditions that can alter the bone healing capacity leading to critical-sized defects resulting in non-union [2]. To meet the growing demand for non-union defect treatment, various bone repair methods have been developed, with autologous bone grafts being considered the gold standard, despite their many adverse effects, including donor-site morbidity, high non-union rate, limited availability and poor structural integrity [3]. Unlike calcium-phosphate (CaP) ceramics, which usually display low strength and brittle behaviour resulting in premature scaffold failure metallic biomaterials are able to support the injured site under load-bearing conditions [4,5]. Stainless steel [6], titanium [7] and cobalt-based alloys [8] are the three main metallic bioinert materials that have been extensively employed for permanent bone replacement [9]. However, their short-term use for fracture stabilization during bone healing remains controversial as conventional metal alloys often lead to stress-shielding and sub-optimal bone formation. In those cases, a second surgery for implant removal is required [10].

In this context, biodegradable metals offer temporary mechanical support for tissue healing and then gradually degrade to provide an optimal environment for blood vessel formation (angiogenesis) and bone regeneration [11,12]. More specifically, Mg-based alloys represent a promising combination of biodegradability, bone healing capacity and mechanical properties [13], closer to those of bone than most other biomaterials [11,14], thus reducing the risk of premature failure [5] and stress-shielding [15]. In addition, Mg, the fourth most abundant cation in the human body [16], does not exhibit common side effects of other metallic biomaterials, such as cytotoxicity, inflammation, allergy or damage to the cardiac and nervous systems [17,18]. However, Mg is one of the most electrochemically active metals, which makes it prone to corrosion [11] associated with high amounts of hydrogen released, leading to the formation of gas cavities *in vivo* [13,19]. Despite the fundamental importance of achieving an optimal corrosion rate, obtaining a controlled and adequate degradation remains challenging when using Mg scaffolds. Several strategies have been investigated to reduce the corrosion rate, including the addition of alloying elements [[20], [21], [22]] and the use of coatings [23,24], but, to date, *in vivo* studies show that Mg alloys are unable to combine durable biomechanical properties with an appropriate corrosion rate and the absence of excessive release of hydrogen leading to gas cavity formation [25]. Therefore, enhanced Mg-based implants produced using innovative manufacturing processes [26,27], resulting in specific architecture, and alloy composition [4,21] have been developed in order to ensure their more successful application in clinical practice.

An assessment of the *in vitro* corrosion pattern of Mg-based scaffolds is fundamental to validate their ability to degrade with an appropriate corrosion rate without releasing excessive amounts of hydrogen. This evaluation has been extensively performed by means of weight loss measurements, hydrogen evolution and electrochemical analysis [[28], [29], [30], [31], [32], [33]], however, although they allow quantification of the corrosion rate, they do not provide details of local morphological changes [34] which may impact tissue growth as the alloy degrades. To address these limitations, the use of X-ray computed tomography (XCT) has emerged, providing a unique 3D insight into material degradation patterns in a non-destructive way [[35], [36], [37], [38]], as well as being able to investigate variations in microstructures derived from morphological changes [14,39,40].

Several authors have pointed out the crucial importance of tuning the corrosion rate with bone regeneration to ensure sufficient mechanical support of the scaffold until bone formation has been completed [4,[11], [12], [13]]. In fact, substantial deterioration of mechanical properties (e.g. Young's modulus, yield stress) is usually observed when pure Mg scaffolds are immersed in simulated body fluid (SBF) *in vitro* [41,42], potentially resulting in premature failure *in vivo*. Such mechanical evaluations are traditionally performed using uniaxial compression or tension [28,32,43,44]; despite the information provided on the overall mechanical behaviour, the correlation between local microcrack formation induced by corrosion and the magnitude of deformation remains unexplored. Favourable local mechanics is crucial for achieving efficient load transfer, as the accumulation of strain locally can affect the overall mechanical properties leading to failure [14,45]. *In situ* mechanics coupled with time-lapsed high-resolution XCT and digital volume correlation (DVC) provides a powerful and unique tool for the experimental computation of 3D full-field strain distribution in bones [[46], [47], [48], [49], [50]], biomaterials [51] and bone-biomaterial systems [45,52]. However, to the authors' knowledge, only one study has investigated the local mechanics and morphological changes of non-corroded Mg-based scaffolds using *in situ* XCT experiments and DVC [14].

Therefore, the purpose of this study is to employ SEM and XCT imaging combined with DVC to evaluate the 3D full-field strain distribution and *in vitro* degradation pattern of an open-porous fluoride coated Mg-based scaffold, produced by liquid phase sintered melt-extracted fibres made of magnesium alloy WZM211 to be used as a treatment for critical-sized bone defects.

## 2 Methodology

### 2.1 Sample preparation

The open-porous Mg-based scaffolds were composed of WZM211 (MgY 2 wt%, Zn 1 wt%, Mn 1 wt%) fibres and further details about its manufacturing process have been extensively reported elsewhere [19,53,54]. Briefly, single short fibres of approximately 4–8 mm in length and 100–250 μm in diameter, were produced by crucible melt extraction (CME) in a high-purity argon-6.0 atm. The fibres were then sintered by liquid phase sintering at 10 K/min until 600 °C, followed by further heating at 3 K/min until 628 °C, held for 20 min. All temperatures were calculated based on differential scanning calorimetry (DSC) analysis on WZM211 in order to retrieve the melting point. The sintering temperature was determined using the PANDAT software package (PanMagnesium, CompuTherm, Madison, USA) [53]. Cylindrical samples (55% porosity) were cut by CNC machines to final dimensions of 10 mm in length and 6 mm in diameter. Finally, all samples were coated with a MgF_2_ layer using a conversion coating method where the fibres were boiled in a sodium hydroxide solution, resulting in a dense magnesium hydroxide layer, followed by immersion in 40% hydrofluoric acid [24,55]. For cleaning, the scaffolds were immersed and agitated in distilled water for 30 s, then rinsed in 100% ethanol for 1 min and dried with compressed air.

### 2.2 In vitro corrosion

To assess dynamic corrosion, samples were placed into the 2 cm^3^ wells of a mechanical stimulation system (MechanoCulture TR, CellScale, Canada) and fully immersed in Hank's balanced salt solution (HBSS Gibco, ThermoFisher, US) circulating at a constant rate of 0.3 mL/min (peristaltic pump P-1, Pharmacia Biotech, US) [43]. The solution inside the wells was continuously refreshed by the flow from a 500 mL tank of HBSS. The accelerated corrosion setup was maintained at 37 °C and 5% CO_2_ and specimens were exposed to dynamic corrosion for 2, 8 and 14 days (n = 3), referred to as Mg2, Mg8 and Mg14, respectively. Additionally whilst undergoing dynamic corrosion 3 samples were subjected to continuous *in situ* cyclic mechanical stimulation up to 30 N (i.e. elastic deformation [14]) at a frequency of 1 Hz [38] to achieve 150000 load cycles, simulating a 2-months postoperative phase [56], over 2 days, referred to as Mg2c. Cyclic mechanical stimulation could only be performed during the first 2 days of corrosion as the samples were completely degraded after 3 days of continuous cyclic loading in HBSS. The pH of the medium was recorded daily (Orion 8103BNUWP ROSS Ultra pH Electrode, Thermo Scientific, US) and found to be constant around 7.46 ± 0.52. After corrosion, the samples were allowed to dry for 1 day at room temperature.

### 2.3 Electron microscopy

The coating layer microstructure and composition were evaluated by scanning electron microscopy analysis (5 kV, SEM, EVO MA10, Zeiss, US) coupled with energy-dispersive X-ray spectroscopy (20 kV, EDX, X-Max 80, Oxford Instruments, UK). Surface observations, as well as cross-sectional examination of the fibres, were carried out to assess the evolution of corrosion microdamage and the coating layer microstructure. The cross-sections were prepared using a water-free protocol [57]. The specimens were first embedded into a low-viscosity resin (EpoThin 2, Buehler, USA). They were then cut to expose transverse sections through the fibres and polished with a polycrystalline diamond suspension (successively 6, 3 and 1 μm) for approximately 5–8 min. A glycerol-based solution was employed as a lubricant. Before imaging, the samples were carbon-coated to improve image acquisition quality (Automatic SEM carbon coater, Agar Scientific, UK).

The thickness of the coating layer was calculated from SEM images of the resin embedded section using Fiji [58]. Four locations per time point were randomly selected at the fibre-coating interface and 10 measurements were performed per location. The fibre-coating interface was determined by visual inspections of the Mg and fluorine (F) EDX maps, but could not be identified in the Mg2c sample due to their greater degree of corrosion which caused deep relief at the interface between the resin and the fibres preventing a sufficiently flat surface to be obtained after polishing.

### 2.4 In situ mechanical testing and XCT images

Prior to *in vitro* corrosion, 3D images of the full pre-corroded specimens were acquired by means of high-resolution XCT (XTH-225, Nikon metrology, UK). 2400 projections were carried out over 360° at 100 kV and 95 μA for a resulting voxel size of ∼25 μm.

Similar parameters were used to conduct *in situ* XCT mechanical testing on corroded Mg-based specimens; each extremity of which was embedded into acetal endcaps using a custom made alignment system in order to minimize end-artefacts [59]. They were then placed in the loading stage (CT500 500 N, Deben Ltd, UK) fitted within the X-ray microscope chamber. *In situ* uniaxial stepwise compression tests were performed with a speed rate of 0.2 mm/min at room temperature. A preload of 10 N was first applied to ensure contact with the compression platens, followed by two repetitive scans, without repositioning, for estimation of the DVC strain uncertainty [60]. Single tomograms were then acquired at 1, 3, 6 and 10% apparent compression. All specimens were allowed to settle for 15 min prior to image acquisition to minimize image artefacts due to stress relaxation [47].

Young's modulus was determined from the stress-strain curves by calculating the slope of the linear regression between σ30 or σ90, corresponding to 30 and 90% of the ultimate stress. The correlation value (R^2^) of the calculated slope was equal or superior to 99% in all cases [61]. The linear regression was then offset by 0.2% to calculate the yield stress.

### 2.5 Image post-processing and morphometry

The 32-bit XCT datasets were reconstructed with VGSTUDIO MAX (VGStudio MAX 2.0.5, Volume Graphics, Germany) and rigidly registered with a correlative metric in Avizo (Avizo 9.7, ThermoFisher Scientific, US) using the first preloaded image as a reference. Each image was cropped to include only the specimen structures in the field of view (∼6 mm^3^) and converted to binary images using Otsu's method [62].

The corrosion rate (CR) was estimated using the binary 3D tomograms and applying equation (1):

(1) $$CR=\frac{\Delta V}{A*\;\Delta t}$$

where *A* was the initial total surface area of the fibres exposed to corrosion (533 ± 58 mm^2^) computed from the 3D XCT images before corrosion using the BoneJ [63] module of Fiji [58], *Δt* the corrosion time (i.e. 2, 8 or 14 days) and *ΔV* the reduction in volume, equal to the difference between the initial pre-corroded volume the remaining volume. Corrosion maps were obtained by subtracting the registered binary image of pre-corroded samples by the corresponding binary image of corroded samples before loading, resulting in the quantification of lost material volume.

Morphometric properties, such as pore size and solid volume fraction, were computed and corresponding 3D maps were produced using the pore network and volume fraction map modules of Avizo.

### 2.6 Digital volume correlation

DVC (DaVis v10.0.5, LaVision Ltd, Germany) was performed between the first preloaded image and those at 1, 3, 6 and 10% apparent compression to compute the 3D full-field third principal strain (εp3) of the scaffolds while being stepwise compressed *in situ*. The DaVis software is based on a local cross-correlation approach operating on the intensity values (grey-level) of the 3D images. Further details of the operating principles have already been reported elsewhere [49,64]. A multi-pass scheme with decreasing sub-volumes, from 110 to 20 voxel [65,66], and 0% overlap [60] was used, followed by vector postprocessing, where sub-volumes with a correlation coefficient below 0.6 were removed. Strain uncertainties were found to be below 400 με in all cases [60,64].

### 2.7 Statistical analysis

Differences in coating thickness and morphometric parameters were analysed by a Kruskal-Wallis test with Dunn-Bonferroni multiple comparison post hoc tests. All statistics were performed using SPSS statistics (SPSS statistics 25, IBM, USA) and the significance level was α = 0.05.

## 3 Results

### 3.1 Electron microscopy

**Fig. 1:**
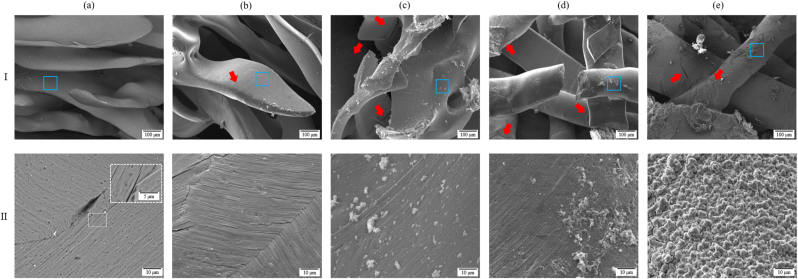
SEM images of the surface of the Mg fibres at (I) 500X magnification and (II) higher 5kX magnification focusing on the coating layer indicated by blue squares in (I) at (a) 0, (b) 2, (c) 2 cyclic, (d) 8 and (e) 14 days of *in vitro* corrosion in HBSS. Red arrows indicate microcrack initiation.

The surface of the pre-corroded fibres showed no apparent microdamage at the macro scale (Fig. 1Ia). However, at the micro level, the coating layer of pre-corroded specimens showed an inhomogeneous covering, highlighted by the presence of coating microcracks of 0.5–1 μm in diameter (Fig. 1IIa). After corrosion, the coating layer displayed a rougher surface contrasting with the smooth surface of the pre-corroded coating. Particle deposition started to occur after 2 days of *in vitro* corrosion in HBSS, along with the initiation of transverse microdamage, that substantially increased after 8 and 14 days, leading to critical fibre failure identified by visual inspections (Fig. 1Id). The application of *in situ* cyclic compression (Mg2c) led to increased particle deposition on the coated surface compared to specimens without cyclic loading (Mg2).

**Fig. 2:**
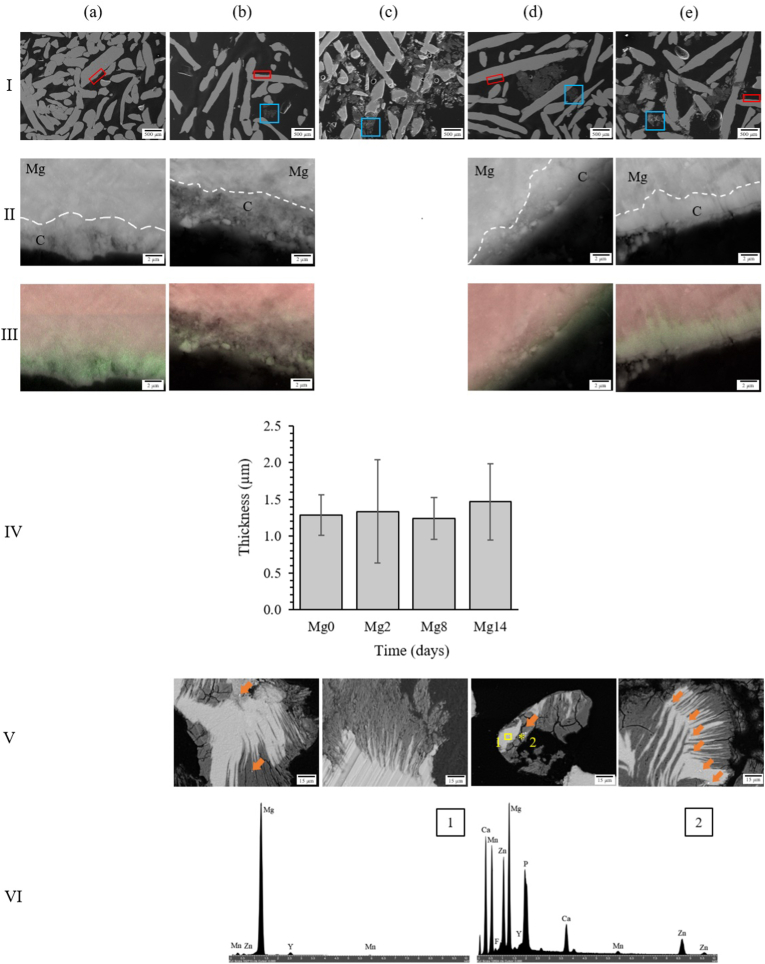
Resin embedded cross-section SEM images of the Mg fibres at (I) 80X magnification, (II) higher 20kX magnification indicated by red squares in (I) targeting the coating/fibres interface highlighted by a dashed white line and (III) its corresponding EDX mapping where Mg is shown in red and fluorine in green at (a) 0, (b) 2, (c) 2 cyclic, (d) 8 and (e) 14 days of *in vitro* corrosion in HBSS. No images could be obtained for Mg2c as the interface between the Mg fibres and the coating layer could not be clearly identified due to their greater degree of corrosion preventing a sufficiently flat surface to be obtained after polishing. (IV) Thickness of the coating layer over time. (V) 1kX magnification indicated by blue squares in (I) targeting corrosion debris, and (VI) EDX spectrum of areas 1 and point 2 delimitated by the yellow square and star in (Vd), investigating the non-corroded area and particle deposition, respectively. Mg., Mg-based alloy; C., coating layer. Orange arrows indicate brighter Ca, P, Zn and Mn-enriched particle deposition.

The resin embedded sections of the samples showed that most of the fibres remained uncorroded with a few localized accumulations of corrosion debris within the pores (Fig. 2I). Distinct textures allowed for visual differentiation between the Mg-based fibres and the coating layer, confirmed by explicit variations of Mg and fluorine (F) content assessed by EDX mapping (Fig. 2II and III). While the Mg fibres displayed a homogenous structure in all cases, the aspect of the coating layer changed over time. First, it appeared more fragmented and less dense than pre-corrosion for Mg2. Then, it was subjected to a redensification process producing a more compact layer after 8 days of *in vitro* corrosion in HBSS. The average thickness of the coating layer was 1.3 ± 0.2 μm and no significant changes were observed over time (Fig. 2IV).

The 2-day cyclic specimens showed a higher volume of corrosion particles trapped between the inner fibres (Fig. 2Ic). The structure of the residual corrosion particles was investigated in more detail using higher magnification (Fig. 2V). Two different Mg phases were identified, with a highly irregular interface, and deep penetration of dark areas (dark grey) into brighter areas (light grey) in the form of filiform corrosion. Intense microcrack propagation occurred within the darker areas, starting from the surface of the fibre and then propagating towards the centre. Severe fragmentation of the outer layer and the formation of debris was observed at 14 days. High-intensity particle deposition was observed after corrosion and their elemental content was analysed by means of EDX examinations (Fig. 2VI) in contrast to the central regions of the fibres. Although Mg remained the main component in both cases, large variations in zinc (Zn) and manganese (Mn) content, which were lower in area 1 than in point 2, were observed. Besides these different proportions of alloying elements, additional chemical elements were found in point 2, with high concentrations of calcium (Ca) and phosphorus (P) whose Ca:P ratio is 1.78 ± 0.14.

### 3.2 Mechanical properties

**Fig. 3:**
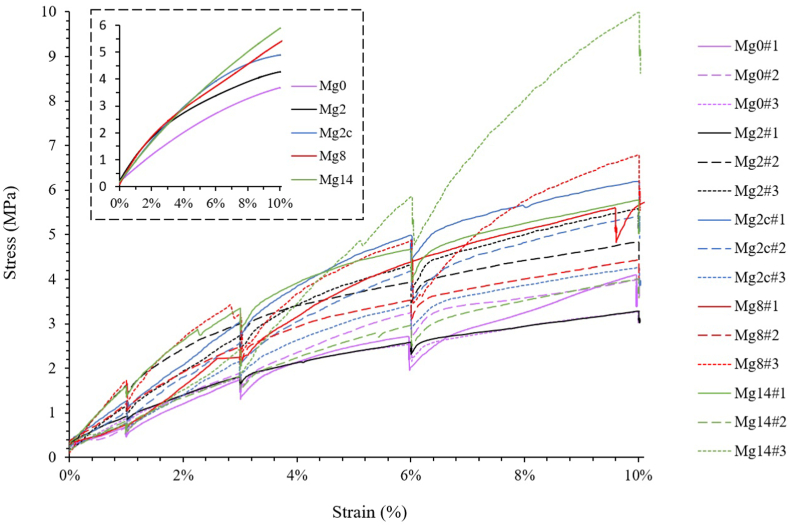
Compressive stress-strain curves of Mg-based scaffolds after 2, 2 cyclic, 8 and 14 days of *in vitro* corrosion, the dashed square contains the average curves of the 3 samples. The data for the non-corroded Mg-based scaffolds (Mg0) were obtained from a previous study [14].

The stress-strain curves for Mg2, Mg2c, Mg8 and Mg14 (Fig. 3) displayed similar trends without obvious distinction between the different immersion times, except for Mg14#3 where the apparent maximum stress reached at 10% compression was twice as high. The stress/strain curves started with a linear elastic region followed, between 2 and 3% compression, by a long strain hardening region where the stress increased up to 10% compression. No critical failure occurred in any of the samples. However, the mechanical properties (i.e. apparent Young's modulus and yield stress) increased with immersion time (Table 1). Mg14 exhibited higher Young's modulus and yield stress (Young's modulus of 89 ± 39 MPa and 110 ± 38 MPa, and yield stress of 2.2 ± 0.6 MPa and 3.0 ± 1.3 MPa for Mg2 and Mg14, respectively). Interestingly, Mg2c showed a Young's modulus closer to Mg2 (89 ± 39 MPa and 86 ± 8 MPa for Mg2 and Mg2c, respectively), but yield stress as high as Mg14 (2.9 ± 0.5 MPa and 3.0 ± 1.3 MPa for Mg2c and Mg14, respectively).

**Table 1:** Mechanical properties of Mg-based scaffolds after 0, 2, 2 cyclic, 8 and 14 days of *in vitro* corrosion. Data are shown as mean ± SD. The data for the non-corroded Mg-based scaffolds (Mg0) were obtained from a previous study [14].

	Mg0 [14]	Mg2	Mg2c	Mg8	Mg14
Young's modulus E (MPa)	68 ± 28 [14]	89 ± 34	86 ± 8	113 ± 37	110 ± 38
Yield stress σ (MPa)	2.1 ± 0.8 [14]	2.2 ± 0.6	2.9 ± 0.5	2.3 ± 0.4	3.0 ± 1.3

### 3.3 Corrosion rate and morphometry

**Fig. 4:**
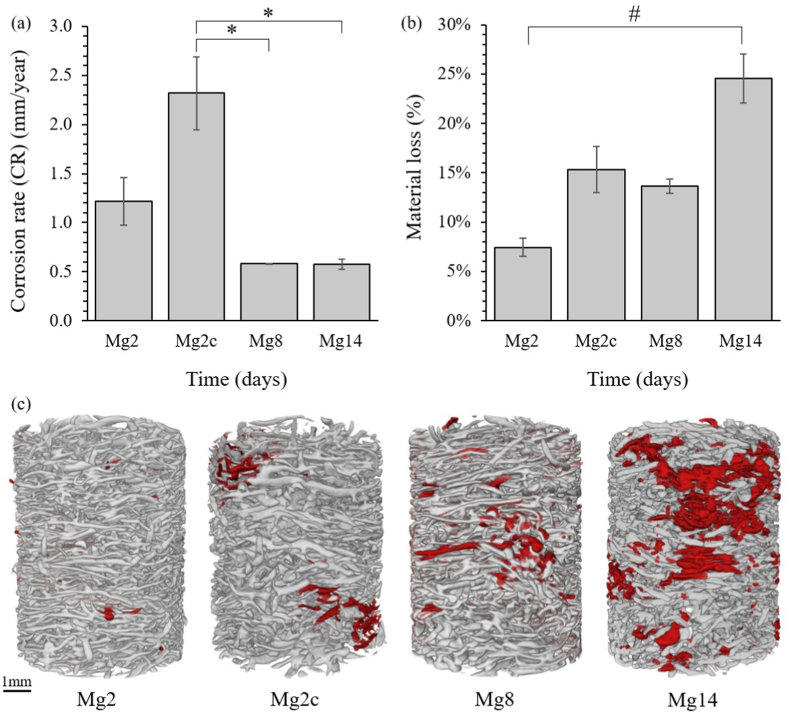
(a) Corrosion rate and (b) material loss volume with immersion time. Data are reported as mean ± SD (n = 3). (c) Reconstructed XCT tomograms for a representative Mg-based specimen. Red areas indicate material loss after *in vitro* corrosion. *p < 0.05 and #p < 0.01 significant differences.

The corrosion rate was higher during the first 2 days of incubation, then decreased, although not significant, with longer immersion time (1.2 ± 0.2 mm/year and 0.6 ± 0.1 mm/year after 2 and 14 days, respectively) reaching a plateau after 8 days where the rate was maintained up to 14 days (Fig. 4a). The fastest corrosion rate (CR) was obtained for Mg2c when *in situ* cyclic compression was applied in combination with dynamic immersion. Although the CR was not significantly different from Mg2, it was significantly higher (p < 0.05) than Mg8 and Mg14. In contrast, the amount of material loss calculated as a volume, increased significantly (p < 0.01) from 2 to 14 days (7.4%–24.5% for Mg2 and Mg14, respectively). Mg2c, although subjected to a shorter corrosion time, achieved a similar material volume loss as Mg8 (Fig. 4b). In all cases, the material volume loss was heterogeneous and occurred in restricted areas mainly located at the periphery of the scaffolds (Fig. 4c).

**Fig. 5:**
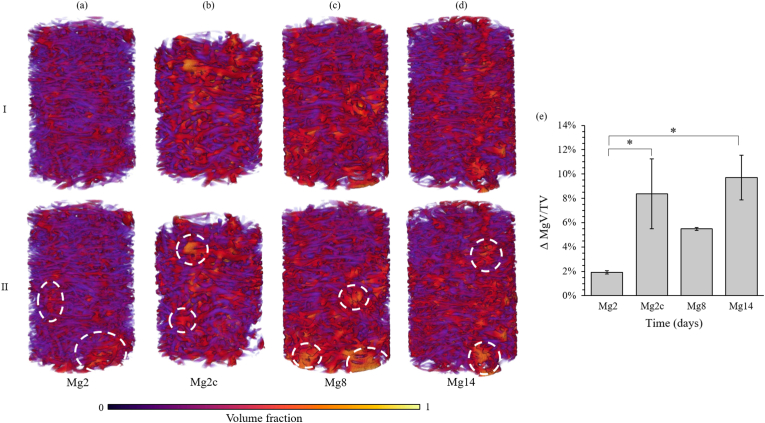
Volume fraction maps for a representative Mg-based specimen (I) before and (II) after (a) 2, (b) 2 cyclic, (c) 8 and (d) 14 days of *in vitro* corrosion. (e) Variation in average volume fraction equal to the difference between the initial and post corrosion values. Data are reported as mean ± SD (n = 3). White ovals indicate local densification after corrosion. *p < 0.05.

The immersion of the Mg-based scaffolds in HBSS solution induced local changes in microstructure. The initial structures displayed heterogeneous local volume fractions ranging from 0.15 to 0.50 (Fig. 5). In all cases, these variations were amplified after *in vitro* corrosion (Fig. 5II), with the occurrence of denser local areas up to 0.70, and were mostly found at the periphery of the scaffolds. Mg14 exhibited a significant (p < 0.05) increase in overall volume fraction (9.7 ± 1.8%) compared to Mg2 (1.9 ± 0.1%, Fig. 5e). Mg2c also presented a significant (p < 0.05) increase in volume fraction (8.4 ± 2.9%), equivalent to that obtained for Mg14.

**Fig. 6:**
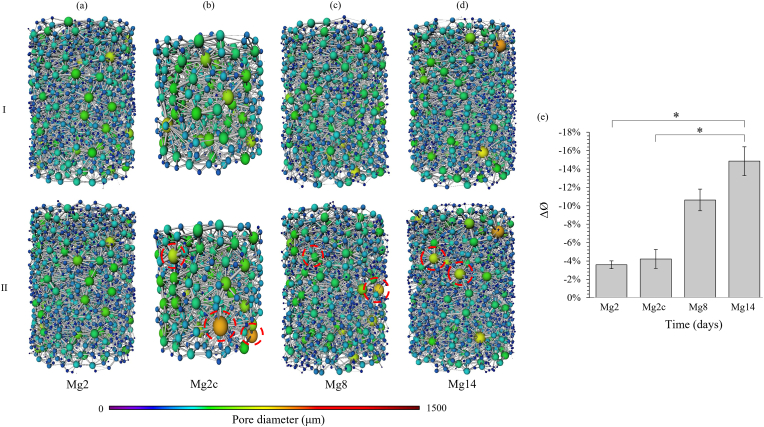
Pore network for a representative Mg-based specimen (I) before and (II) after (a) 2, (b) 2 cyclic, (c) 8 and (d) 14 days of *in vitro* corrosion. (e) Variation in average pore diameter equal to the difference between the initial and post corrosion values. Data are reported as mean ± SD (n = 3). Red ovals indicate local pore diameter increase after corrosion. *p < 0.05.

The pore network also showed a heterogeneous distribution before corrosion with a majority of smaller pores in the centre of the scaffold (300 μm) and larger pores (600 μm) at the periphery (Fig. 6I). Despite local enlargement of the external pores from 450 μm before corrosion to 700 μm after 14 days, the corrosion led to a significant (p < 0.05) overall reduction in pore diameter (Fig. 6e), which became more pronounced with longer corrosion times (reduction of 3.6 ± 0.4% and 14.9 ± 1.6% for Mg2 and Mg14, respectively).

### 3.4 Digital volume correlation

**Fig. 7:**
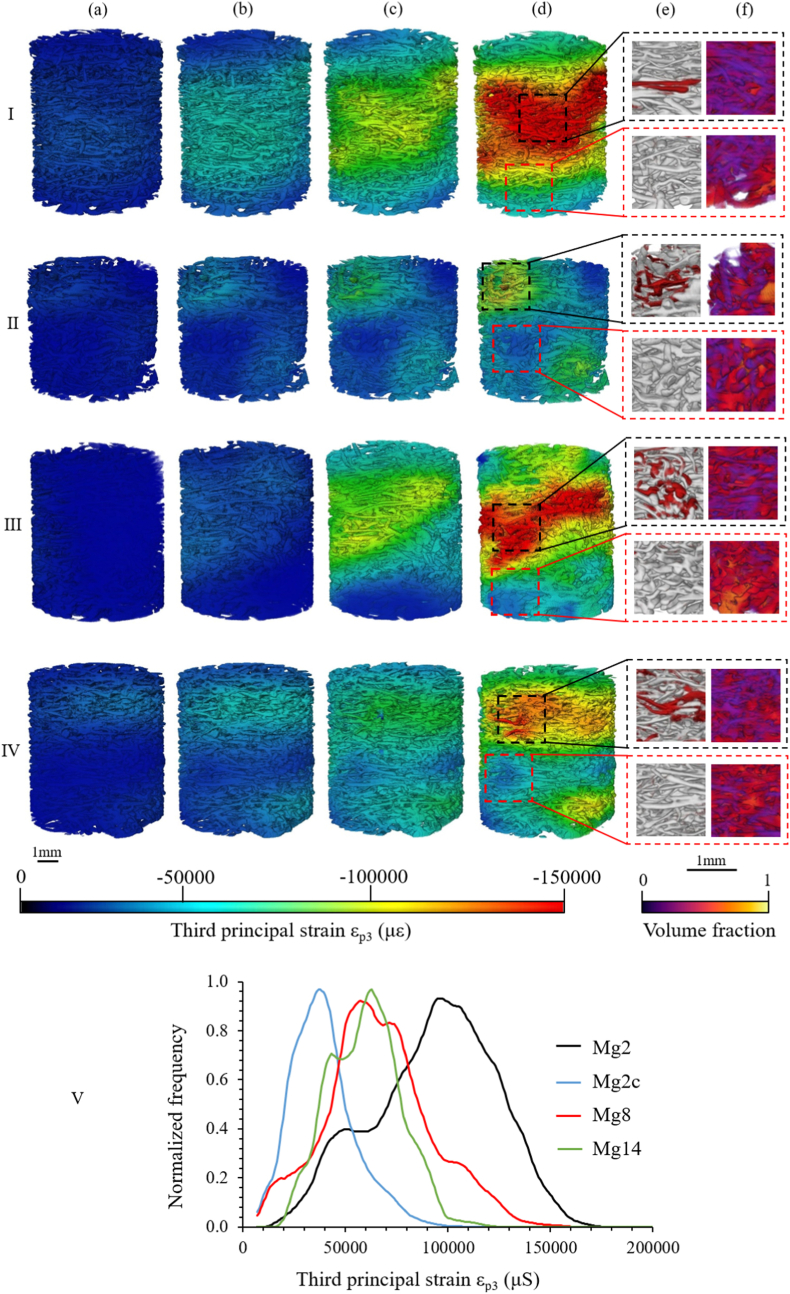
Full-field third principal strain (ε_p3_) distribution at (a) 1%, (b) 3%, (c) 6% and (d) 10% for a representative Mg-based specimen at (I) 2, (II) 2 cyclic, (III) 8 and (IV) 14 days of *in vitro* corrosion. (V) Normalized histogram of the average third principal strain after apparent yielding at 10% compression of the three specimens tested per corrosion time. Normalization was performed by dividing the number of counts by the mode (i.e. most frequent value) of each histogram. (e) Reconstructed XCT image where red parts indicate material loss and (f) volume fraction maps associated with high-strain (dotted black square) and low-strain (dotted red square) regions.

Local third principal strain (ε_p3_) distributions computed by DVC are shown in Fig. 7. In all cases, the strain distribution was heterogeneous with large variations ranging locally from −10000 με to −150000 με at 10% compression. However, the apparent high-strain region was evenly distributed in the core of the scaffold for Mg2 and Mg8, while Mg2c and Mg14 multiple areas of higher strains were located at the edges of the structures. A gradual increase in strain was observed across the compression steps in all cases (−4617 ± 2008 με and −63230 ± 17954 με in average for 1% and 10%, respectively). Interestingly, lower strain accumulation occurred with longer corrosion times, as well as in Mg2c (Fig. 7V), where the average ε_p3_ strain decreased from −91000 ± 6361 με to −60093 ± 2414 με and −40791 ± 1321 με after yielding at 10% compression for Mg2, Mg14 and Mg2c, respectively.

A local structural analysis was carried out by examining the selected volume of interest of the preload XCT tomograms corresponding to the high (>−100000 με) and low-strain regions (≤−100000 με) at 10% compression in order to explain their distribution. In all cases, greater material loss and a lower volume fraction (Fig. 7e and f) were observed in the high-strain region compared with the low-strain region (0.3 and 0.5 average volume fraction in the high and low-strain regions, respectively).

## 4 Discussion

The aim of this study was to investigate the *in vitro* degradation pattern of a novel Mg-based alloy produced by liquid phase sintering. The effects of degradation of the alloy were studied using SEM and XCT. *In situ* XCT mechanics, in combination with DVC, was used to measure the full-field third principal strain (ε_p3_) distribution within the apparent elastic and post-yield regimes in order to evaluate the influence of the corrosion process on the mechanical and morphological performance of the scaffolds. Degradation was initially rapid with a corrosion rate of 1.22 ± 0.24 mm/year after 2 days and this then significantly decreased from day 2 to day 8 (0.58 ± 0.01 mm/year) to remain unchanged until day 14 (Fig. 4a). Although this trend is commonly observed when Mg-based materials are immersed in corrosion media [37,41,67,68], the corrosion values reported in this study were lower than those obtained with pure uncoated Mg [29,67], fluoride-coated [36] and various Mg alloys [19,69,70]. However, external parameters such as the composition of the solution employed [69,[71], [72], [73]] or the flow rate [74,75] can substantially influence the corrosion rate and should be considered when comparing studies. In fact, Johnston et al. [75] observed a higher corrosion rate, associated with a more uniform corrosion pattern, under dynamic flow than in static immersion. Similarly, an acceleration of the degradation was induced by the application of *in situ* cyclic compression in simulated body fluid (SBF) due to the amplification of stress corrosion by continuous and repetitive loading [76,77]. The corrosion protocol implemented in this study combined a dynamic flow of HBSS able to buffer the environment surrounding the Mg-based scaffold and simulating physiological pH lowering with *in situ* cyclic loadings conducive to fast corrosion. Thus, the lower corrosion rates observed from 2 to 14 days, demonstrated the ability of this unique Mg-based scaffold to effectively resist *in vitro* degradation with most of the fibres remaining intact after immersion (Fig. 2I) and nearly 75% of the initial structure retained (Fig. 4b) resulting in an overall conservation of structural integrity. Moreover, although the highest corrosion rate obtained for Mg2c (2.32 ± 0.37 mm/year) was comparable to the *in vivo* values of uncoated Mg–6Zn, Mg–2Sr and Mg–2Y–1Zn (WZ21) for which hydrogen gas cavities were observed [29,68,78], the corrosion rates have been extensively reported to be much faster *in vitro* than *in vivo* [19,29,70,79]. It can therefore be expected that the corrosion rate of the present scaffold would be slower *in vivo* achieving a low level of degradation without the formation of local gas cavities [19,24,70].

The fluoride coating still covered the fibres at 14 days (Fig. 2II) but suffered from substantial changes in structure up to 14 days of *in vitro* corrosion in HBSS. The cross-sectional sections first revealed a dissolution of the coating layer after 2 days of corrosion compared to the pre-corroded state, followed by a re-densification probably due to the precipitation of calcium-phosphate crystals that tended to stabilize the coating and form a more protective corrosion layer. Similar deposition of round-shaped particles on magnesium alloys has already been observed and attributed to calcium phosphate (CaP) particles [41,80,81], with a Ca:P ratio close to 1.67, suggesting hydroxyapatite formation. Due to the presence of fluoride in the coating layer of the alloy, it is most likely that fluorapatite crystals have formed that can explain the slight divergence in Ca:P ratio (1.78 ± 0.14) obtained in this study [82]. Therefore, besides providing a protective layer that slowed down the corrosion process (Fig. 4a), the fluoride coating promoted the deposition of calcium-phosphate crystals, crucial for the mineralization of bone tissue *in vivo* and indicative of an osteoconductive surface.

While most of the fibres remained uncorroded, a localized accumulation of corrosion debris, trapped between the central fibres of the scaffold, appeared from day 2 and increased over 14 days (Fig. 2I). SEM cross-section images revealed a fragmented coating layer with long filiform pitting corrosion penetrating through the fibres that were aligned with microcracks (Fig. 2V). Considering the heterogeneous and stripe-like shape of the corrosion pattern of a few microns thickness, we hypothesise that the coating microcracks found on the surface of the coating layer initiate the corrosion process that continues and penetrates the fibres, forming corrosion microcracks that lead, with longer immersion time, to complete fibre failure (Fig. 1Id).

The corrosion process was characterised by material loss which increased with immersion time (7.4 ± 0.9% and 24.5 ± 2.5% of material loss from the initial volume at 2 and 14 days, respectively), and was mainly located at the periphery of the scaffold. The outermost pores of the structure, which were initially larger than the inner pores, underwent further enlargement from 430 to 750 μm on average (Fig. 6). However, despite this external local increase in porosity, the structure was subjected to overall densification after corrosion, reducing the inner pore diameter (reduction of 3.6 ± 0.4% and 14.9 ± 1.6% at 2 and 14 days, respectively) and increasing the volume fraction (increase of 1.9 ± 0.1% and 9.7 ± 1.8% at 2 and 14 days, respectively) (Fig. 5), principally due to the accumulation of corrosion debris. The three-dimensional arrangement of the scaffold represents a crucial factor in ensuring clinical success as this may enhance cell migration, invasion of blood vessels and bone formation. Leading to enhanced osteoconductive properties [83,84]. Chang et al. [84] have shown, by varying the porosity and pore size of hydroxyapatite blocks implanted *in vivo*, that highly mineralized lamellar bone tissue was obtained with a specific pore size between 300 and 500 μm. XCT tomograms of this Mg-based scaffold showed morphometric similarity to trabecular bone as we reported previously [14], and although the average pore diameter, which measured 550 ± 10 μm, was lower than trabecular bone (1000 μm) [85], it was within the range identified by Chang et al. [84]. In the present study, the overall porosity (Fig. 5) computed after 14 days of immersion (48 ± 7%) still fell within the range of trabecular bone [86,87] and the average pore size (302 ± 46 μm at 14 days) within the optimal range of 300–500 μm. Therefore, although corrosion induced structural changes over time, the resulting Mg-based structure maintained a 3D morphology conducive to bone formation.

The Mg-based scaffolds exhibited similar mechanical behaviour regardless of immersion time, characterised by a linear elastic region followed by a long strain hardening without any apparent stress reduction or critical failures in the deformation range tested (i.e. up to 10% compression) (Fig. 3). Similar trends have widely been observed with Mg-based scaffold under mechanical compression testing before [14,[88], [89], [90], [91], [92]] and after [28,32,41,93] corrosion. Gibson et al. [94] described the mechanical behaviour of cellular solids and found that the hardening phase is usually followed by a densification,whereby the pores start to collapse. This densification phase was also observed by Jiang and He [88] for pure Mg 45–55% porous structures (up to 60% compression at 1 mm/min) that displayed a regular increase in stress as also described by Hedayati et al. [28] and Li et al. [93] after 1 and 7 days of corrosion. Therefore, it was expected that the Mg-based scaffolds used in this study would withstand high deformation without global failure up to 14 days of corrosion and that beyond the deformation range tested, the hardening phase will be followed by a densification phase.

Despite a significant material loss, higher apparent mechanical properties (Table 1) were observed after 14 days of corrosion compared to the initial samples or after 2 days of corrosion [14]. This variation correlated with the overall increase in volume fraction (Fig. 5e) and the reduction in pore diameter (Fig. 6e) due to the accumulation of corrosion debris in the structure (Fig. 2). Thus, it appeared that the overall densification of the structure and the morphological changes caused by the degradation process were involved in the enhancement of mechanical performance. Similarly, an increase in mechanical properties occurred with Y-RE-Mg (WE43) alloys immersed in SBF [93], Al–Zn–Mg (AZ63) and Mn–Mg (M2) alloys [28] in PBS during the early stages of corrosion (2 days and 5 h, respectively), however, this was followed by a reduction of Young's modulus and yield stress. In both cases, the temporary rise in mechanical properties was attributed to the accumulation of corrosion particles. In contrast, most studies investigating the influence of corrosion on the mechanical performance of Mg-based materials reported a constant decrease in mechanical properties when subjected to compression [32,41,42], tension [31] and three-point bending [33]. However, although various chemical compositions were explored, none of these Mg-based scaffolds were coated, suggesting that the enhanced mechanical behaviour of the present scaffold may be due to its fluoride coating which reduced the corrosion rate. In addition, the entangled design of this structure (Fig. 4c) tended to retain the corrosion debris and preserved the mechanical integrity even after 14 days of corrosion. Nevertheless, while this densification of the structure seemed to be particularly advantageous in compression, it may be less beneficial in tension or torsion and should be further investigated.

The third principal strain (ε_p3_) accumulation decreased on average from 2 to 14 days (Fig. 7V) but was higher than measured in the non-corroded samples (−26243 ± 7870 με, average ε_p3_ at 10% compression) [14]. This suggested that during the first two days, when the corrosion rate was faster, the trade-off between the external material loss and internal accumulation of corrosion products seemed unfavourable resulting in lower mechanical performance, while after 2 days the structure regained strength and load-bearing properties similar to that seen prior to corrosion. The strain distribution also displayed a more heterogeneous pattern after corrosion (Fig. 7), while regions of high strain were largely spread over the structure before corrosion [14], they were more localized in specific areas after corrosion. Local morphometric observations revealed a higher volume fraction (Fig. 7f) as well as higher material loss (Fig. 7e) in the high-strain regions than in the low-strain regions, indicating that local variations in morphometric properties led to higher local deformation. It appeared therefore, that the corrosion pattern, which caused structural changes, was actively and directly involved in the overall strain distribution and mechanical behaviour of the Mg-based scaffold.

Corrosion studies on biodegradable metals are usually performed using static [19,28,[30], [31], [32], [33],^69^,^70^,^93^] or dynamic [37,[41], [42], [43], [44]] flow, simulating physiological fluids *in vivo*, but the impact of repetitive loading remains unexplored. Only a few studies have combined *in vitro* dynamic immersion with *in situ* cyclic loadings to investigate the degradation pattern of Mg-based scaffolds [38,76,77], but an intense loading frequency (10 Hz) up to premature failure as well as a higher flow rate (65 mL/s) than physiological intraosseous blood flow rate [95] were used. The present corrosion protocol was designed in order to best approximate *in vivo* corrosion conditions by employing an appropriate flow rate and cyclic frequency averaging 150000 cycles after 2 days *in vitro*, which is comparable to the number of steps taken by the patients during a recovery period of approximately 2–3 months [56]. The addition of cyclic compression increased the corrosion rate, that in turn accelerated material loss, to levels comparable to those of 8 days (Fig. 4), apparent densification (Fig. 5) and accumulation of corrosion debris (Fig. 2). The increase in corrosion rate can be explained by the stresses applied to the alloy during cyclic loading which induced local disruptions of the coating layer through the formation of fatigue microcracks [76]. The cyclic movement of the scaffolds can also be responsible for the accelerated detachment of weakly bonded corrosion products that tend to reduce the protective corrosion layer usually formed during corrosion.

Under constant *in situ* cyclic loading (i.e. 225000 cycles) simulating more than 2 months of post-surgical recovery [56], the scaffolds lasted for 3 days before being fully degraded. The scaffold is therefore expected to maintain the mechanical integrity of the injured site *in vivo* for the first months after surgery. During this period, bone callus will form and begin to mineralize, helping to compensate for the loss of mechanical support due to scaffold degradation [45]. In addition, the influence of soft tissue, which cannot be simulated *in vitro*, will also favour an efficient and protective effect. Alternatively, a possible solution to extend the mechanical integrity of the Mg-based scaffolds could be obtained by a modified coating.

The structural changes caused enhanced mechanical behaviour, including higher Young's modulus and yield stress (Fig. 3) without critical failure and lower ε_p3_ accumulation (Fig. 7), thus preserving mechanical integrity despite a corrosive environment. In addition, these mechanical values fell within the range of trabecular bone reported in literature; 0.01–2 GPa for Young's modulus and 0.2–80 MPa for yield stress [96]. This resemblance to trabecular bone and its ability to deform without fracturing would be beneficial in preventing premature failure *in vivo* and would lead to a more uniform strain distribution, providing a better mechanical stimulus for bone regeneration [97,98]. Furthermore, the strain range employed to deform the Mg-based scaffolds after corrosion exceeded, by one order of magnitude, the physiological strain of bone tissue generated during daily activities such as walking or running (500–2000 με) [99], suggesting that, in theory, the corroded scaffold would also endure any high-intensity impact.

## 5 Conclusion

The purpose of this study is to evaluate the *in vitro* degradation pattern of a unique Mg-based scaffold composition, produced by liquid phase sintering and its influence on morphological and mechanical performance. In particular, the combination of high-resolution XCT images and DVC has allowed characterization, for the first time, of the full-field third principal strain in corroded Mg-based scaffolds. The fluoride coating efficiently protected the Mg-based scaffold from severe degradation, resulting in a relatively low *in vitro* corrosion rate and preservation of the structural integrity after 14 days of corrosion. The internal accumulation of corrosion debris appeared to led to enhanced mechanical properties and lower third principal strain accumulation under compression, where the distribution was mainly driven by localized material loss and decreased volume fraction. In addition, the optimal load transfer and resulting morphological parameters would tend to favour bone regeneration *in vivo*. Ultimately, the investigated porous Mg-based scaffolds provide a bone replacement, capable of sustaining mechanical loads *in situ* during the postoperative phase allowing new bone formation to be initially supported as the scaffold resorbs.

## Submission declaration

We the undersigned declare that this manuscript is original, has not been published before and is not currently being considered for publication elsewhere.

## Ethical approval statement

The authors state that no animal or cell experiment has been conducted which needs a specific approval.

## CRediT authorship contribution statement

**Roxane Bonithon:** Conceptualization, Methodology, Formal analysis, Investigation, Writing – original draft, Writing – review & editing, Visualization. **Colin Lupton:** Investigation, Resources, Writing – review & editing. **Marta Roldo:** Conceptualization, Resources, Writing – review & editing, Supervision. **Joseph Nicholas Dunlop:** Investigation, Resources, Writing – review & editing. **Gordon William Blunn:** Conceptualization, Resources, Writing – review & editing, Supervision, Funding acquisition. **Frank Witte:** Conceptualization, Methodology, Resources, Writing – review & editing, Supervision, Funding acquisition. **Gianluca Tozzi:** Conceptualization, Methodology, Resources, Supervision, Funding acquisition.

## Declaration of competing interest

The study was partially funded by Biotrics bioimplants and Frank Witte is an employee of the company. All the other authors have no conflict of interest to declare.
