# Supplementary material for: Open-porous magnesium-based scaffolds withstand in vitro corrosion under cyclic loading: A mechanistic study
Source: Bioact Mater. 2022 Apr 29;19:406–17. doi: 10.1016/j.bioactmat.2022.04.012 (PMC9062748; doi:10.1016/j.bioactmat.2022.04.012)
Supplement: Multimedia component 1 [file mmc1.docx]

## Supplementary material


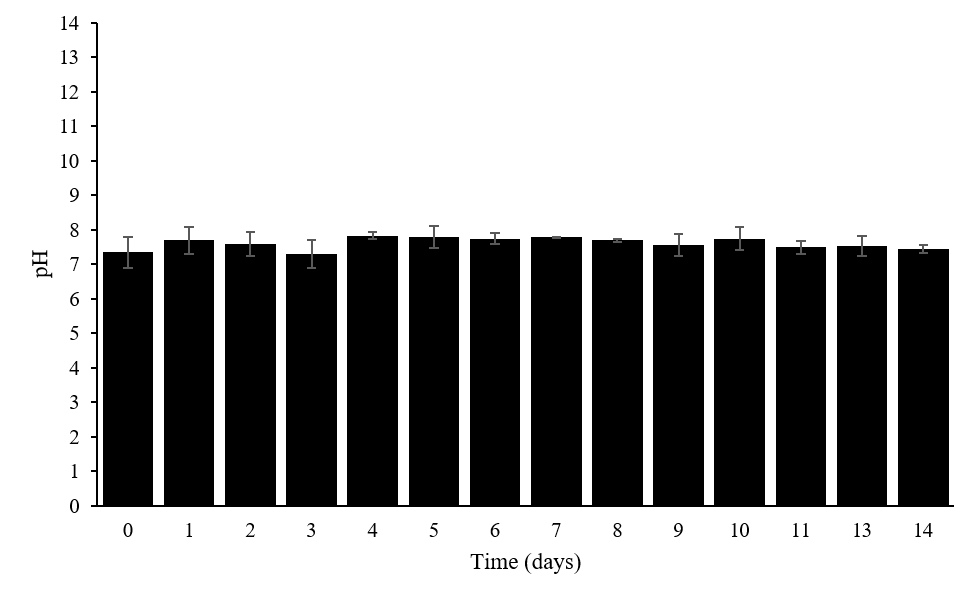


Fig. S1. PH over time during *in vitro* corrosion.


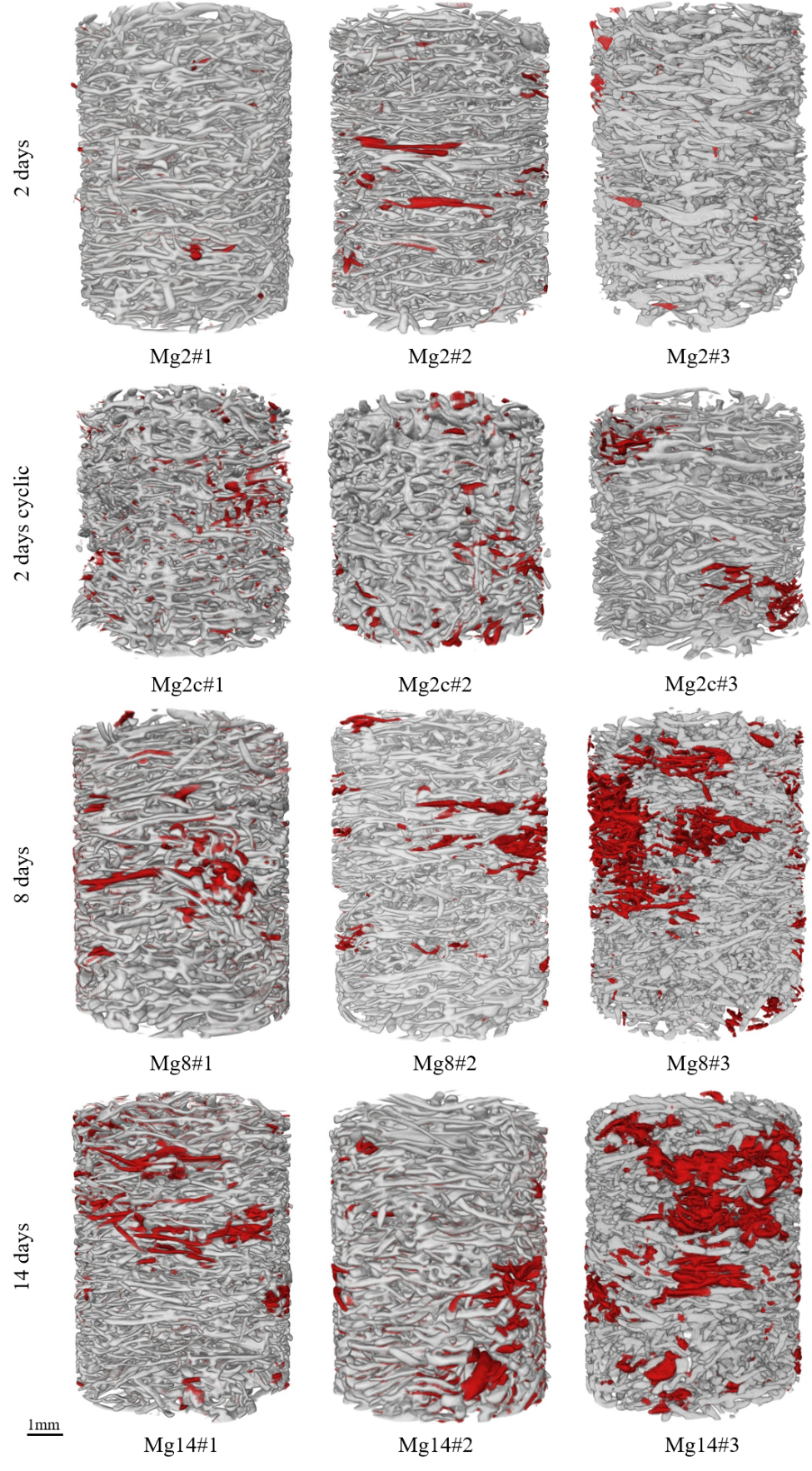


Fig. S2. Reconstructed XCT tomograms after 2, 2 cyclic, 8 and 14 days of *in vitro* corrosion. Red parts indicate corroded material.


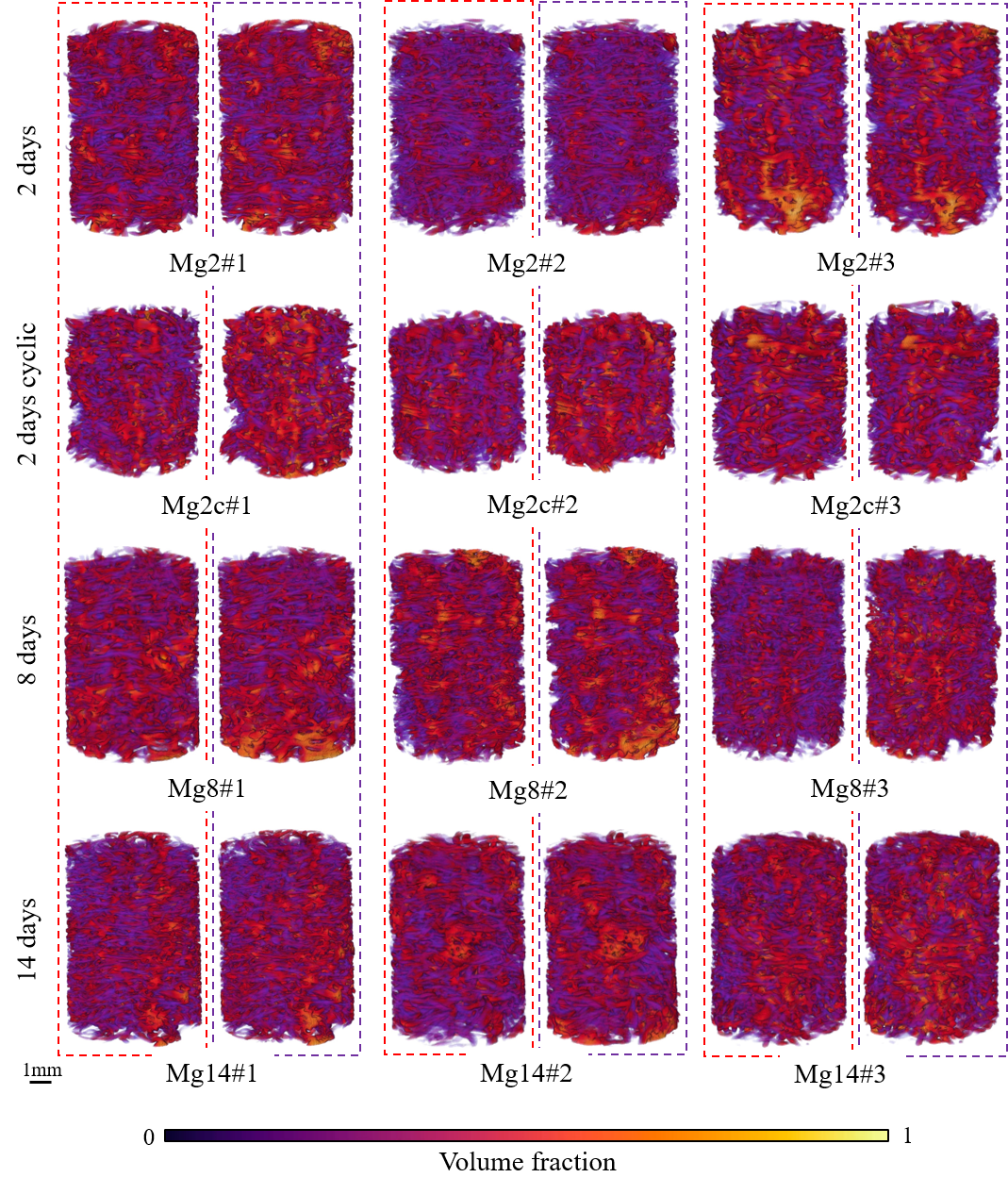


Fig. S3. Volume fraction maps before (red dashed squares) and after 2, 2 cyclic, 8 and 14 days of *in vitro* corrosion (purple dashed squares).


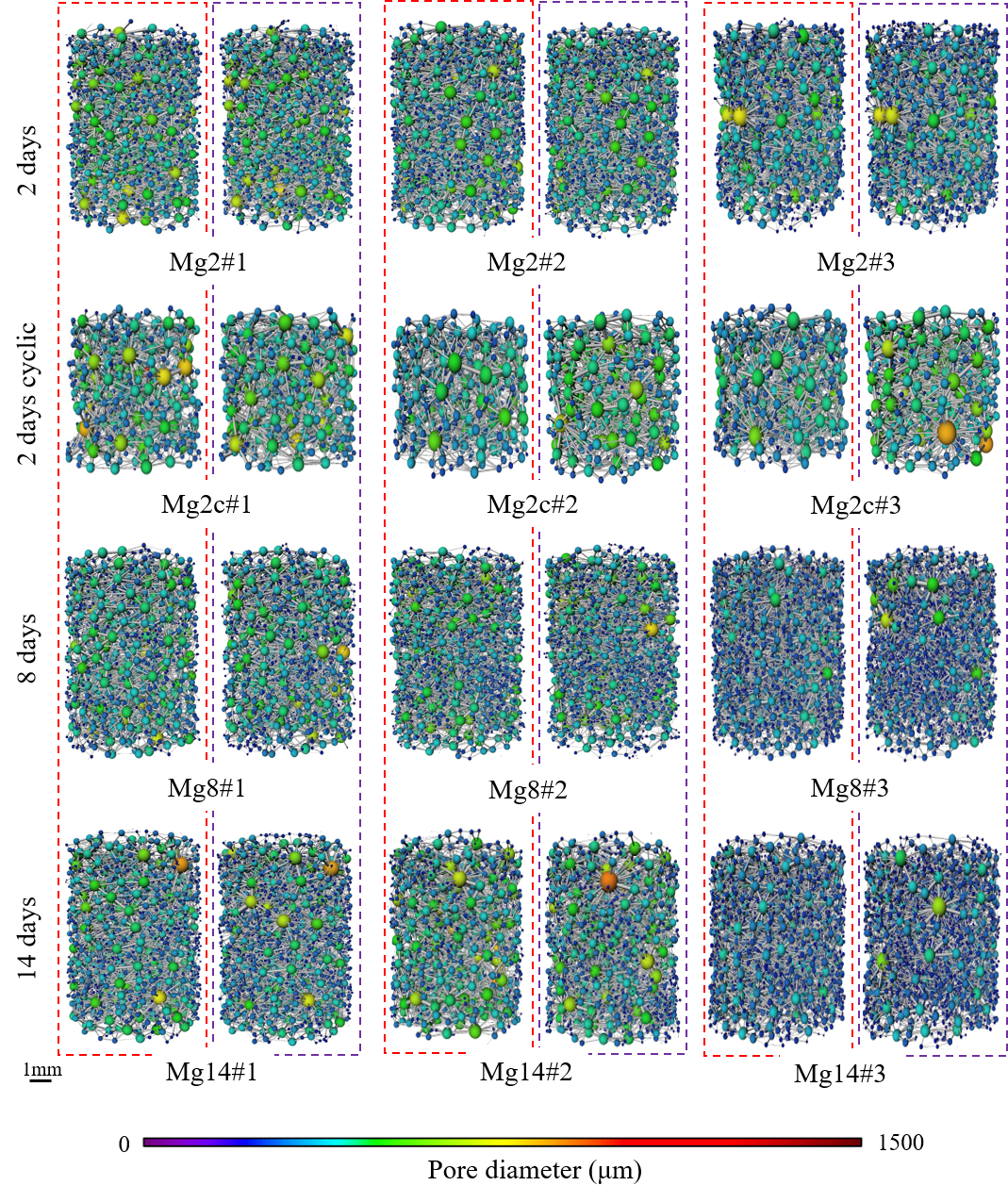


Fig. S4. Pore network before (red dashed squares) and after 2, 2 cyclic, 8 and 14 days of *in vitro* corrosion (purple dashed squares).


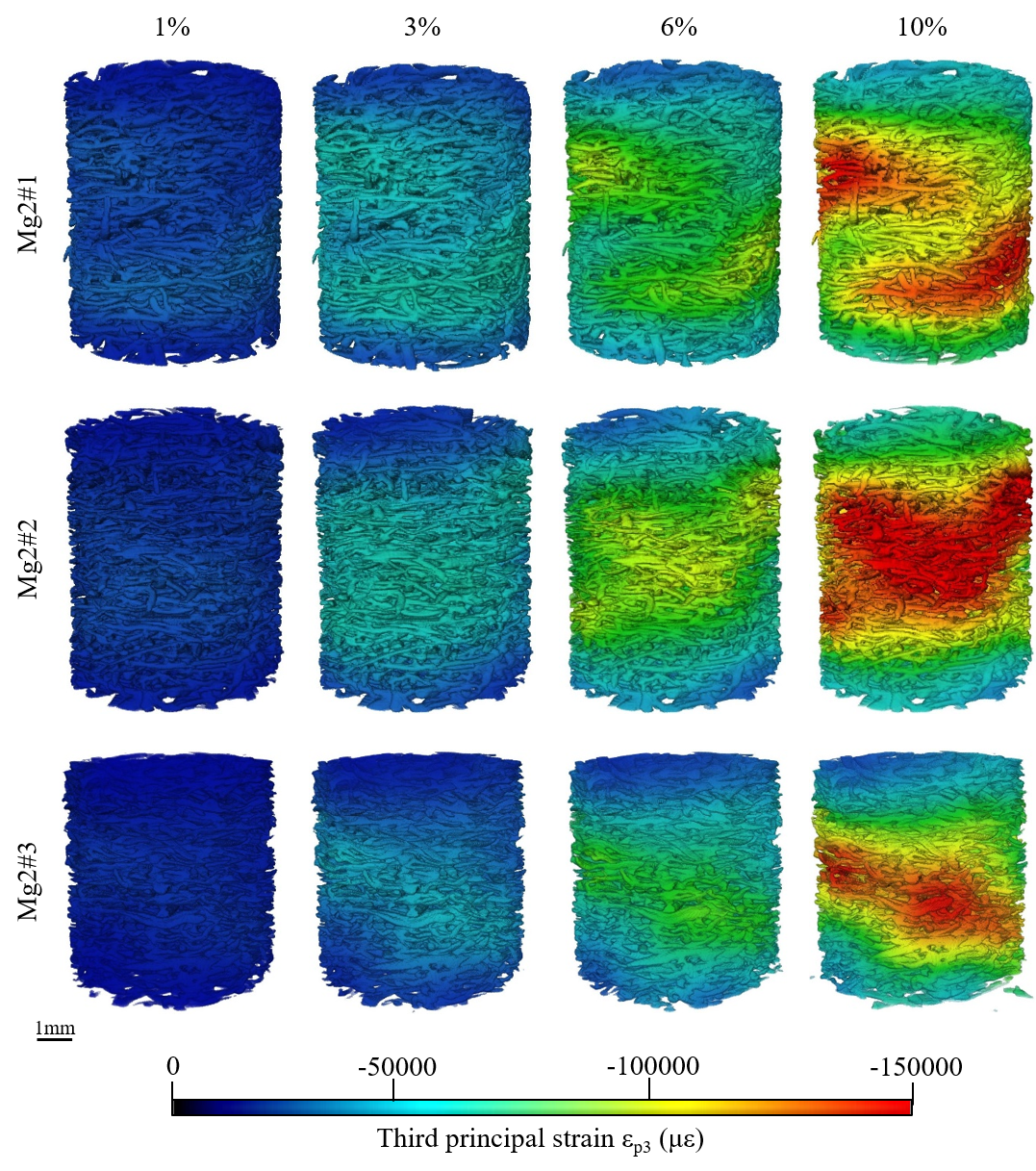


Fig. S5. Full-field third principal strain (εp3) distribution at 1%, 3%, 6% and 10% after 2 days of *in vitro* corrosion.


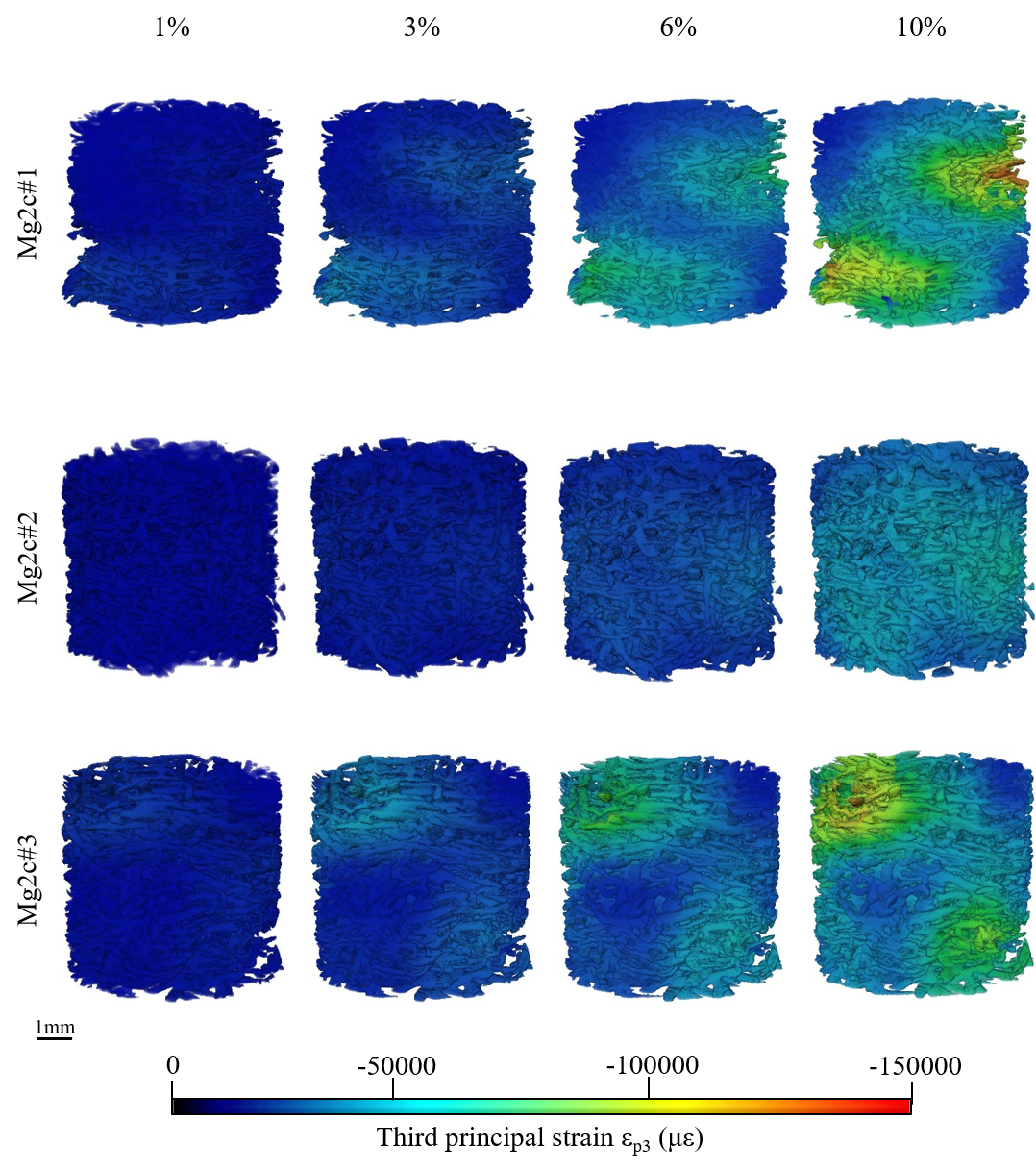


Fig. S6. Full-field third principal strain (εp3) distribution at 1%, 3%, 6% and 10% after 2 days of *in vitro* corrosion combined with cyclic *in situ* compression.


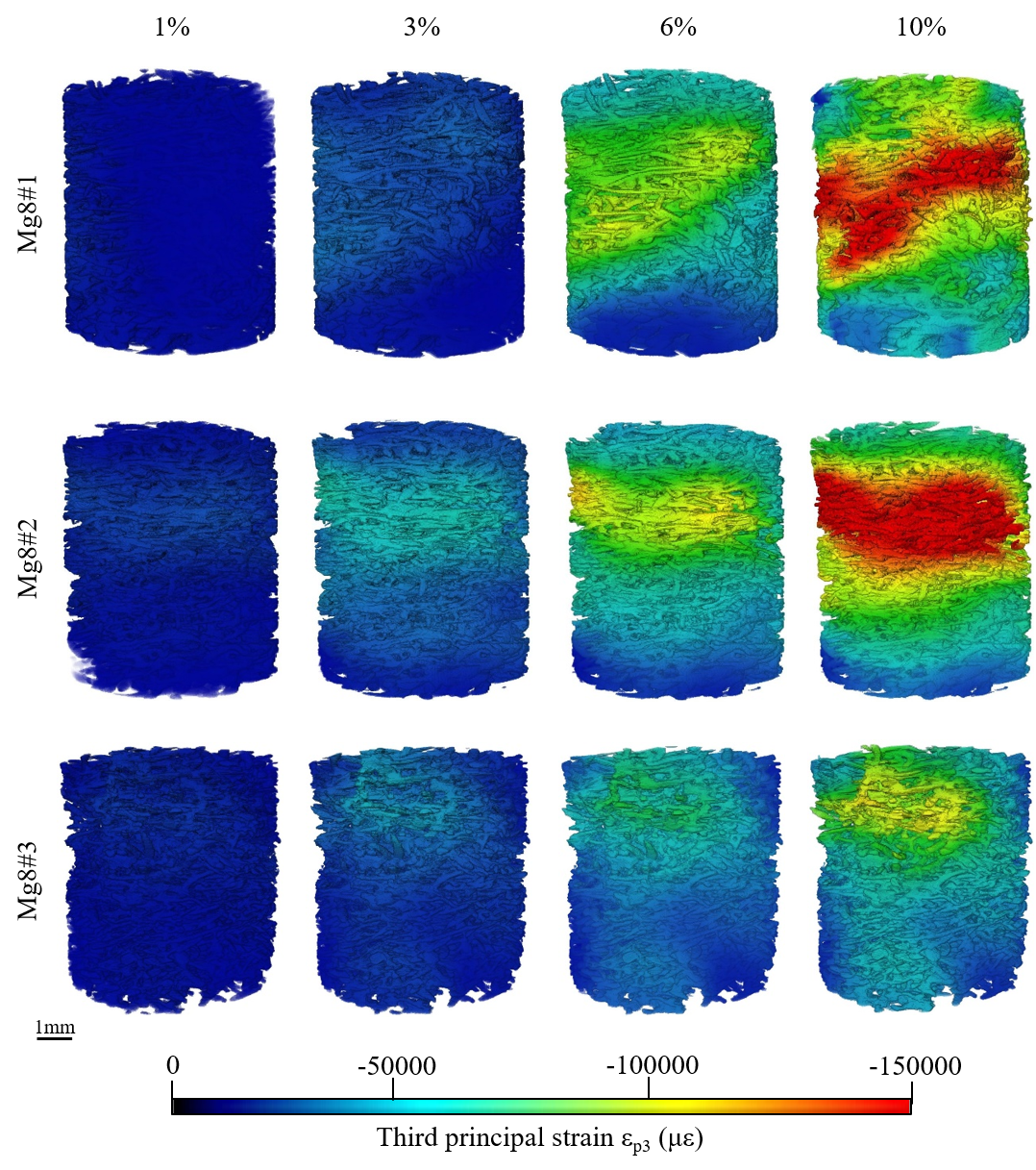


Fig. S7. Full-field third principal strain (εp3) distribution at 1%, 3%, 6% and 10% after 8 days of *in vitro* corrosion.


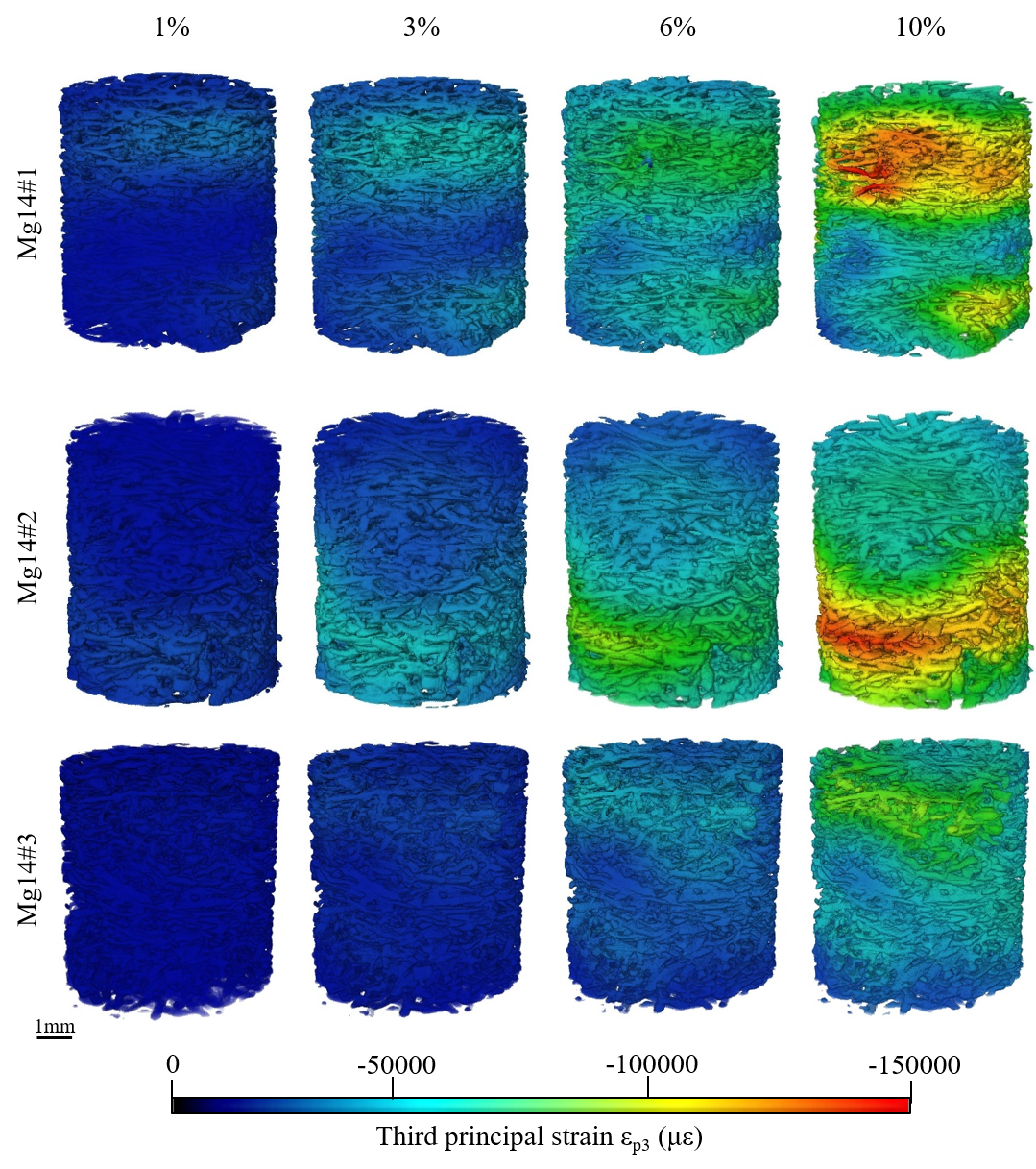


Fig. S8. Full-field third principal strain (ε_p3_) distribution at 1%, 3%, 6% and 10% after 14 days of *in vitro* corrosion.
